# Probe Cytotoxic and Oxidative Stress Effects of Nanoplastics on Caco-2 Cells: Insights from Raman Spectroscopy and Machine Learning

**DOI:** 10.3390/s26175375

**Published:** 2026-08-25

**Authors:** Bryan Gustafson, Negar Kosari, Emily Brothersen, Morgan Mosher, Anhong Zhou

**Affiliations:** Department of Biological and Chemical Engineering, Utah State University, Logan, UT 84322-4105, USA; bryancg32@gmail.com (B.G.); n.kosari@usu.edu (N.K.); emily.brothersen@usu.edu (E.B.); morgan.christensen@usu.edu (M.M.)

**Keywords:** nanoplastics, Caco-2 cells, Raman spectroscopy, apoptosis, machine learning, resveratrol

## Abstract

Nanoplastics and microplastics have become an increasing ecological and health concern due to their widespread presence in the environment and food chain. In this study, Caco-2 cells were used as an in vitro model of the human intestinal epithelium to investigate the cytotoxic effects of polystyrene nanoparticles and microparticles of varying sizes, concentrations, and surface modification. Oxidative stress and apoptosis were evaluated following particle exposure. Raman spectroscopy, combined with machine learning analysis, was employed to detect and characterize biochemical alterations in the cells. Several peak ratios were selected to cluster data depending on size. A correlation between apoptosis and both concentration and Raman score was established highlighting its potential as a non-invasive tool to monitor nanoparticle-induced cellular damage. Resveratrol pretreatment reduced some of these harmful effects of amine-modified nanoplastics, reducing reactive oxygen species generation. This study demonstrates the potential of combining Raman spectroscopy and machine learning to assess the cytotoxic effects of micro- and nanoplastics and identifies protective strategies such as antioxidant pretreatment.

## 1. Introduction

Nanoplastics (NPs) and microplastics (MPs) are a rapidly growing ecological and health concern due to their widespread prevalence [1]. The effect of NPs or MPs primarily relates to cellular response to reactive oxygen species (ROS) but also includes interactive effects with other cytotoxic agents [2,3,4,5,6]. Of particular interest in this work is the effect on mammalian intestinal cells due to the regular, unintentional consumption of NPs in the typical Western diet. Estimates of human ingestion of NPs or MPs range from 74 thousand to 29 billion particles annually [1,7]. A human blood sample can even contain 1.6 µg of plastic per mL [8].

Polystyrene is commonly used in household consumer products, including food packaging and handling items. It was selected as the focus of this study for several reasons: PS nanoparticles are among the most extensively characterized in the nanotoxicology literature, making them a well-established model system for benchmarking new analytical methods; they are commercially available in highly monodisperse, size-controlled formulations essential for isolating size-dependent effects; and our current work shows that the cell-Raman spectra did not exhibit the interference background peaks from polystyrene, making us focus on the analysis of biomolecule peaks from the cells under different PS exposure conditions. Prior in vitro and in vivo studies have shown several biological effects of polystyrene NPs and MPs, including oxidative stress, inflammation, apoptosis, lysosomal malfunction, and mitochondrial dysfunction [9,10]. The size and type of particle play a major role in the severity of biological effects [11]; for instance, the surface charge of particles even show a dramatic impact on those biological effects [12]. While the concentrations used in this study (50–500 µg/mL) exceed current estimates of environmental exposure, they are consistent with concentrations widely adopted in in vitro nanotoxicology literature and are necessary to elicit measurable cellular responses within the timeframe of cell culture experiments. Such suprathreshold concentrations are commonly employed to establish dose-response relationships and identify mechanisms of toxicity that may become relevant under chronic low-dose exposure scenarios or in populations with elevated plastic consumption. Similarly, the acute exposure durations used here (24–48 h) are a deliberate starting point: short-term exposures allow mechanistic endpoints such as ROS generation and apoptosis to be isolated and attributed to specific particle properties without the confounding effects of cell adaptation, proliferation, or passage that accompany longer cultures [3,13,14,15].

Raman spectroscopy has emerged as a powerful, non-destructive analytical technique for investigating the molecular composition and biochemical changes within biological systems [16,17,18]. Advances in noise suppression and signal-to-noise ratio improvements enable more sensitive biomolecular detection, highlighting its potential for label-free, chemically specific, high-resolution identification of micro- and nanoplastics in complex environmental and biological matrices [19,20].

In the context of nanotoxicology, Raman spectroscopy offers significant advantages by allowing real-time monitoring of cellular responses to nanoparticle exposure [21,22,23]. By correlating spectral features with biological endpoints such as cell viability and apoptosis with the aid of machine learning techniques, Raman spectroscopy can serve as a sensitive tool to elucidate the mechanisms of nanoparticle-induced toxicity at the molecular level.

While ROS production is a normal byproduct of cellular respiration, excessive cellular ROS can lead to mitochondrial dysfunction, DNA damage, and eventually mitochondria-dependent apoptosis and necrosis. Resveratrol, a naturally occurring polyphenolic compound, which can be found in a variety of common dietary fruits and herbs, has shown positive effects in reducing ROS, apoptosis, and restoring mitochondrial function under cellular ROS stress [24]. The protective qualities of resveratrol at the cellular level can be explained by its capability to either directly neutralize ROS or by enhancing the expression of genes responsible for normal cellular ROS regulation. Such response includes enzymes such as heme oxygenase catalase, glutathione peroxidase, and superoxide dismutase, or increasing the level of glutathione, which plays a crucial role in maintaining cellular redox balance [25]. Resveratrol has also been shown to upregulate mitophagy, or recycling of damaged mitochondria, which is another mechanism by which resveratrol increases cellular longevity [26].

Resveratrol has been studied as a protective agent against several oxidative stressors, including hydrogen peroxide, ethyl alcohol, and diesel exhaust particles [27,28,29,30]. Several studies have also investigated nanoplastic-induced ROS stress in vitro [31,32] and in model organisms such as *C. elagans* [33]. What appears to be lacking, however, is the application of resveratrol as a protective agent against the negative effects of nanoplastics.

In this study, Raman spectroscopy was employed to investigate the biochemical alterations in human intestinal epithelia Caco-2 cells, a commonly used in vitro model for intestinal barrier, following exposure to polystyrene nanoparticles and microparticles. While previous work has applied Raman spectroscopy and machine learning to characterize oxidative stress in cancer and immune cells [34,35], the present study extends this framework to nanoplastic-induced cytotoxicity in intestinal epithelial cells, a biologically relevant model of dietary uptake. Specifically, we systematically compare four dimensionality-reduction algorithms (PCA, t-SNE, UMAP, and PHATE) applied to the same Raman dataset to assess their ability to resolve size- and concentration-dependent biochemical changes. Furthermore, the Raman score derived from PCA-LDA posterior probabilities is applied here for the first time to nanoplastic toxicology, providing a continuous, single-cell index of cellular damage that correlates with apoptosis measurements from conventional flow cytometry. The goal was to identify molecular fingerprints associated with cytotoxicity and to assess whether spectral signatures could serve as predictive markers to reduce reliance on endpoint assays. The protective effect of resveratrol against nanoplastic-induced damage was then evaluated.

## 2. Materials and Methods

### 2.1. Cell Culture

As a simplistic model of the human gut, Caco-2 cells (ATCC, Cat. No. HTB-37) were grown according to ATCC guidelines. Cells were thawed in a 37 °C water bath and cultured in Eagle’s Minimum Essential Medium (EMEM, ATCC Cat. No. 30-2003, Manassas, VA, USA) with 10% *v*/*v* Fetal Bovine Serum (FBS, Fisher Scientific, Cat. No. A5256701, Ciudad de México, Mexico) and 1% *v*/*v* penicillin/streptomycin (Millipore Sigma, Cat. No. P4333, Saint Louis, MO, USA). Cells were grown in T75 flasks in a 37 °C incubator with 5% CO_2_ and passaged every 3–5 days. All experiments were performed between cell passage 4 and passage 16. All cell manipulations were performed under a Level 2 Biosafety Cabinet. Caco-2 cells were grown in a monolayer to confluency and transferred to 6-well plates at 3 × 10^5^ cells/well prior to ROS and Viability assays. Caco-2 cells were obtained from frozen stocks previously preserved in EMEM +10% *v*/*v* Dimethyl Sulfoxide (DMSO, Millipore Sigma, Cat. No. D1435, Saint Louis, MO, USA) + 10% FBS and frozen in liquid nitrogen vapor. Cells were preserved after 3 passages.

### 2.2. Antioxidant Treatment and Polystyrene Particles

Caco-2 cells were grown to 60–80% confluency in 6-well plates and treated with resveratrol (Sigma Aldrich, Cat. No. V900386, Saint Louis, MO, USA) at specific concentrations prior to treatment with NPs. The antioxidants were dissolved in DMSO. The particles used for this project are polystyrene nanoplastics (PS NPs) (Polysciences, Inc., Warrington, PA, USA) with sizes of 50 nm, 100 nm amine modified, and 500 nm. A physicochemical characterization of all three types of particles in cell culture medium (EMEM + 10% FBS) at 200 g/mL, including hydrodynamic diameter, were measured by DynaPro NanoStar (Wyatt Technology Corporation, Santa Barbara, CA, USA) with software Dynamics version 7.1.7.16. Zeta potential was measured using a Zeta Plus (Brookhaven Instrument Corporation, Holtsville, NY, USA) with BIC Zeta Potential Analyzer version 5.59 software.

### 2.3. Flow Cytometry

ROS and sample viability were measured using CytekBio’s Muse Flow Cytometer. This instrument is capable of analyzing a variety of cell characteristics according to their commercially available kits. Used in this experiment were the Annexin V & Dead Cell kit and the Oxidative Stress kit (Cytek Biosciences, Hayward, CA, USA). Both kits use fluorescent markers conjugated to proprietary antibodies to detect cellular markers for ROS response or apoptosis. For both kits, cells were trypsinized and transferred to microcentrifuge tubes for centrifugation. They were then processed according to the kit instructions. Each treatment condition was performed in at least three independent replicates (*n* = 3).

### 2.4. Raman Spectroscopy

Raman spectroscopy was performed on separate samples prepared identically to those tested by flow cytometry. The Raman spectra were measured on a Renishaw inVia Raman spectrometer using the WiRE 3.4 software interface (Renishaw, Wotton-under-Edge Gloucestershire, UK) connected to a Leica microscope (Leica Microsystems, Buffalo Grove, IL, USA), equipped with a 785 nm laser and focused through a 63× -NA 1⁄4 0.90 water immersion objective (Leica Microsystems, Wetzlar, Germany). The calibration peak for the spectrometer with silicon mode at a static spectrum was 520.5 ± 0.1 cm^−1^. Caco-2 cells were cultured on MgF_2_ (United Crystals Co., Port Washington, NY, USA) and imaged in 1× phosphate-buffered saline solution (PBS). Raman spectra were collected between 450 and 1800 cm^−1^, unless otherwise indicated, for one accumulation of 10 s laser exposure in static mode. Due to the time-consuming nature of Raman spectroscopy, samples of different conditions were prepared at staggered time points such that the treatment times for each sample were roughly identical. Raman data was exported from WiRE 3.4 as .txt files and processed via Python 3.11 script to remove cosmic rays, subtract the baseline signal, and array the data for analysis [36].

### 2.5. Data Processing

Raman data was exported from WiRE 3.4 as .txt files and processed via Python script to remove cosmic rays, subtract the baseline signal, and normalize to [0,1]. Principal component analysis (PCA) was applied to the normalized data to identify the peaks contributing most strongly to the principal components for subsequent single-peak analysis. The PCA score was also calculated using linear discriminant analysis (LDA) for classification. To assess model generalizability and guard against overfitting, LDA classification was evaluated using leave-one-out cross-validation (LOOCV), in which each spectrum was held out in turn as a test sample while the model was trained on the remaining spectra. Classification accuracy reported here reflects LOOCV performance [37,38]. As part of the LDA classification analysis, posterior probability can also be calculated simultaneously. Moreover, the posterior probability was further converted to a Raman score by utilizing the following equation [34]:

$$S=log\frac{p}{1-p}$$

where *p* is the posterior probability and S is the Raman score. This transformation, the logit function, is applied because posterior probabilities from LDA yield highly asymmetrical distributions that are poorly suited for direct comparison across conditions [39]. Applying the logit linearizes the probability values along a continuous scale, enabling the degree of individual cell response to be scored and compared across treatment groups. The score thus provides a continuous, single-cell index of biochemical perturbation rather than a binary group assignment, which is particularly valuable for capturing the heterogeneity of population cellular responses within a treatment group [40]. Although Raman spectra were collected from different cells in different Petri dishes, the cells were from the same treatment group. Individual spectra were used as the unit of analysis for Raman score comparisons, and Raman score results show the general heterogeneity for each treatment group and their shifts across these treatment groups. Raman data are analyzed by other machine learning algorithms: t-distributed stochastic neighbor embedding (t-SNE), principal component analysis (PCA), uniform manifold approximation and projection (UMAP), and potential of heat-diffusion for affinity-based trajectory embedding (PHATE). Each of these algorithms are applied to the same datasets to determine the method of grouping the data that provides the clearest distinction among treatment conditions [41,42,43,44,45,46]. A grid search was completed to determine the hyperparameters for each algorithm. The hyperparameters were optimized for the greatest normalization mutual information (NMI). To determine the clustering efficacy of the methods, both the NMI and the silhouette score were calculated.

## 3. Results

### 3.1. Cytotoxicity of Nanoparticles

The hydrodynamic diameter and zeta potential of all three types of particles (PS50, PS100NH2, PS500) in cell culture medium (EMEM + 10% FBS) at 200 g/mL before and after 24 h are provided in the Supplementary Materials (Figure S1 and Table S1). The results indicate that particle properties remained stable after 24 h, suggesting minimal aggregation under the experimental conditions used.

**Figure 1:**
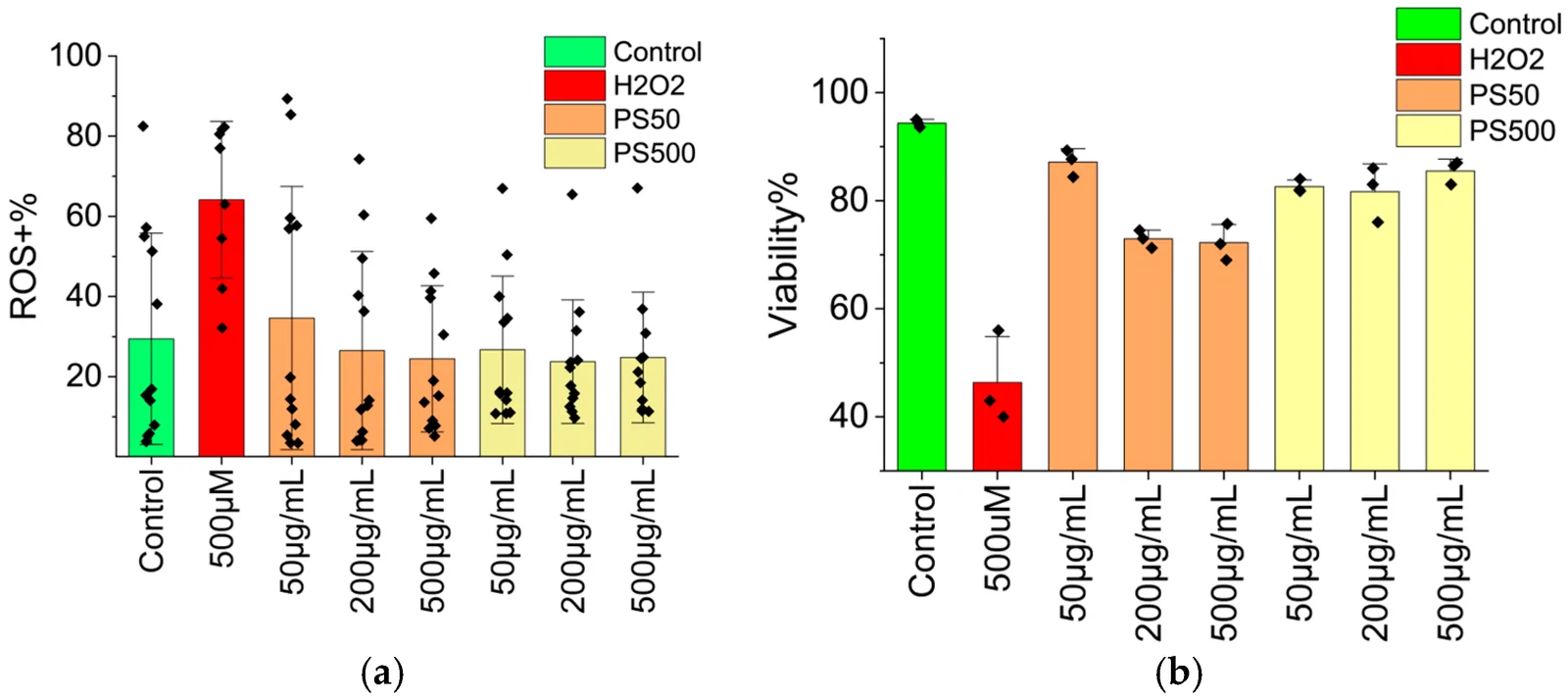
ROS (**a**) and viability (**b**) of Caco-2 cells treated with plain polystyrene particles (PS50, PS500) after 24 h treatment at different sizes and concentrations: 50 µg/mL, 200 µg/mL, 500 µg/mL.

To measure the cytotoxic effects of polystyrene particles, two different sizes were used, which represent 50 nm plain polystyrene nanoparticles (NPs) (PS50) and 500 nm plain microplastics (MPs) (PS500). The results of this experiment are shown in Figure 1a,b. Each type of nanoplastic was used at concentrations 50 µg/mL, 200 µg/mL, 500 µg/mL. Non-normalized ROS and Viability values obtained after 24 h treatment with ROS and Viability of Caco-2 cells treated with plain polystyrene nanoparticles. A t-test was performed between each solvent used for each nanoplastic concentration, and against negative control. In this experiment, PS500 did not show any statistically significant effect of the polystyrene particle concentration on ROS or viability in the particle concentration range of 50 µg/mL to 500 µg/mL. However, at concentrations of 200 and 500 µg/mL, PS50 reduced the percentage of live cells.

While some nanoparticles appear to have minimal impact on overall cell viability, a more detailed analysis of apoptosis profiles reveals potential early apoptotic changes. This suggests that certain nanoparticles may induce cellular stress responses that do not immediately result in cell death but may still disrupt cellular homeostasis [46,47].

**Figure 2:**
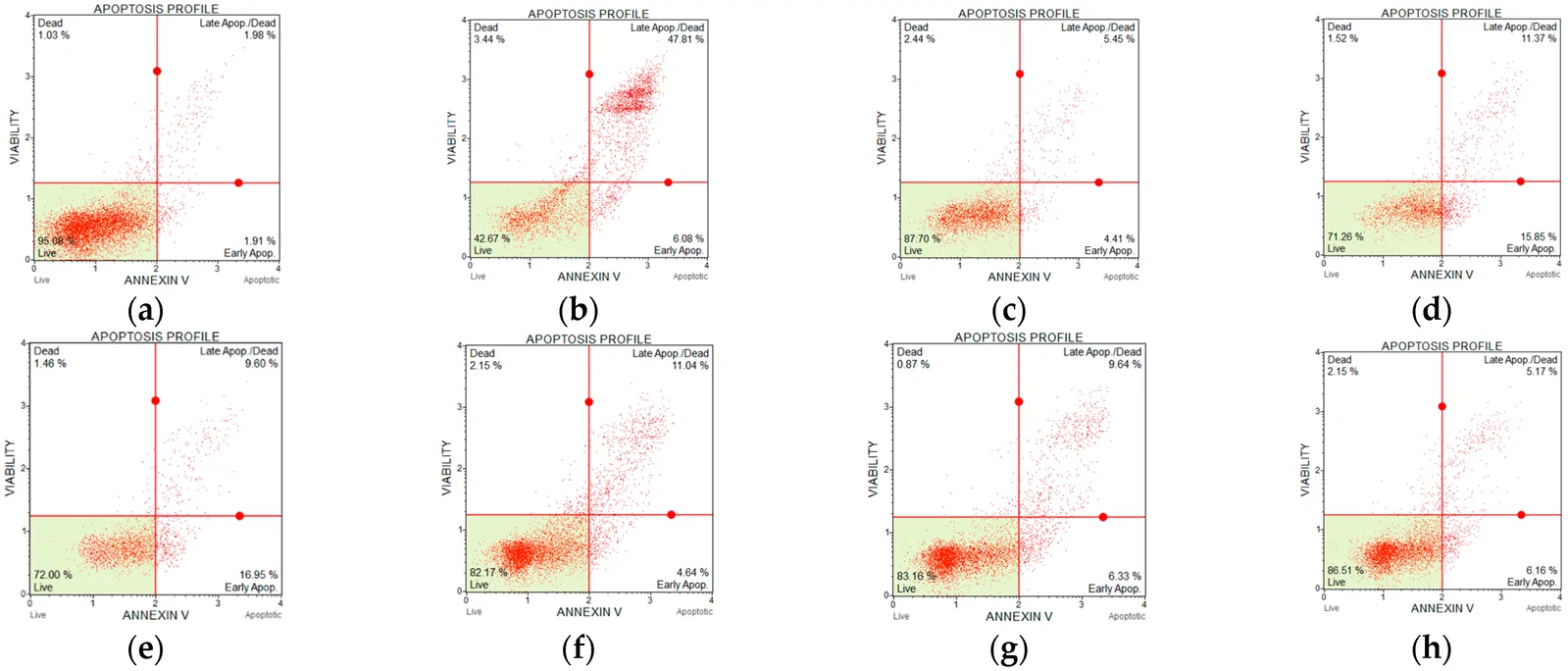
Apoptosis profile of Caco-2 cells treated with PS50 or PS500 after 24 h treatment at different concentrations: control (**a**), 500µM H_2_O_2_ (**b**), 50 nm-50 µg/mL (**c**), 50 nm-200 µg/mL (**d**), 50 nm-500 µg/mL (**e**), 500 nm-50 µg/mL (**f**), 500 nm-200 µg/mL (**g**), 500 nm-500 µg/mL (**h**). These flow cytometry results are extended from Figure 1b.

Figure 2 illustrates these cell apoptotic profiles corresponding to Figure 1b, highlighting the differences in early and late apoptosis across treatment groups.

**Figure 3:**
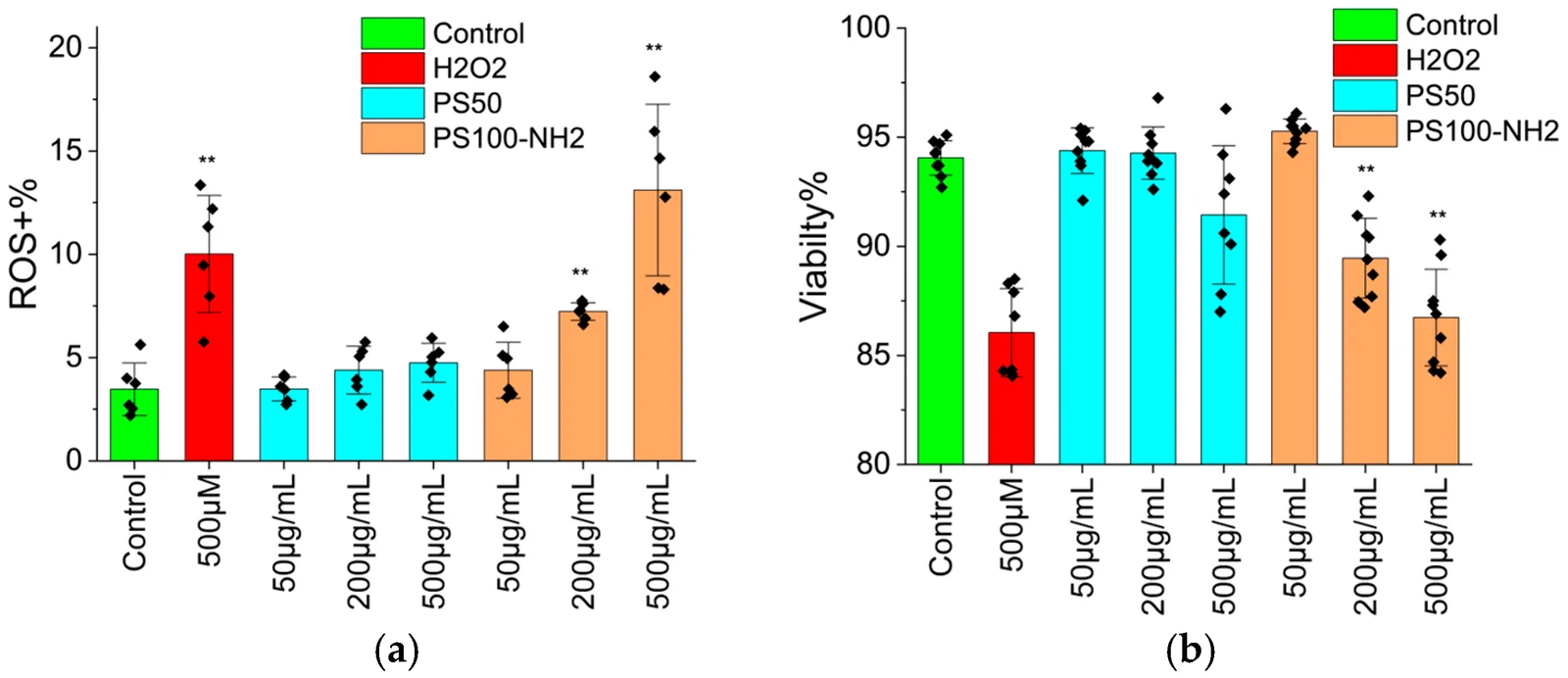
ROS (**a**) and viability (**b**) of Caco-2 cells treated with 50 nm plain polystyrene nanoplastics (PS50) and 100 nm amine-modified polystyrene nanoparticles (PS100NH2) after 24 h treatment at different concentrations: 50 µg/mL, 200 µg/mL, 500 µg/mL. ** indicates significant difference (*p* < 0.05).

After determining that plain polystyrene had no statistically significant effect on ROS using this method, amine-modified polystyrene was tested and compared to plain polystyrene. Due to unavailability of 50 nm amine-modified beads from commercial sources, 100 nm amine-modified beads (PS100NH2) were used instead of 50 nm. Still, previous studies found in published research have shown no significant difference in the biological effects of 50 nm (PS50) and 100 nm (PS100) polystyrene [47,48]. Figure 3 shows the effects of plain polystyrene NPs compared to amine-modified polystyrene, NPs for 50 µg/mL, 200 µg/mL, 500 µg/mL, non-normalized ROS, and Viability of Caco-2 cells treated for 24 h with plain and NH2-modified polystyrene nanoparticles. A t-test was performed between each sample and negative control (*p* < 0.05). H_2_O_2_ is used as a positive ROS control, with a treatment time of 8 h. These data show significant cellular damage in the presence of amine-modified NPs, including major ROS damage, as compared to virtually no detectable change in ROS or viability in the groups treated with plain PS NPs. The apoptosis profile of PS100NH2 can be found in Figure S2.

### 3.2. Cell Raman Spectra Analysis of Cellular Oxidative Stress

**Figure 4:**
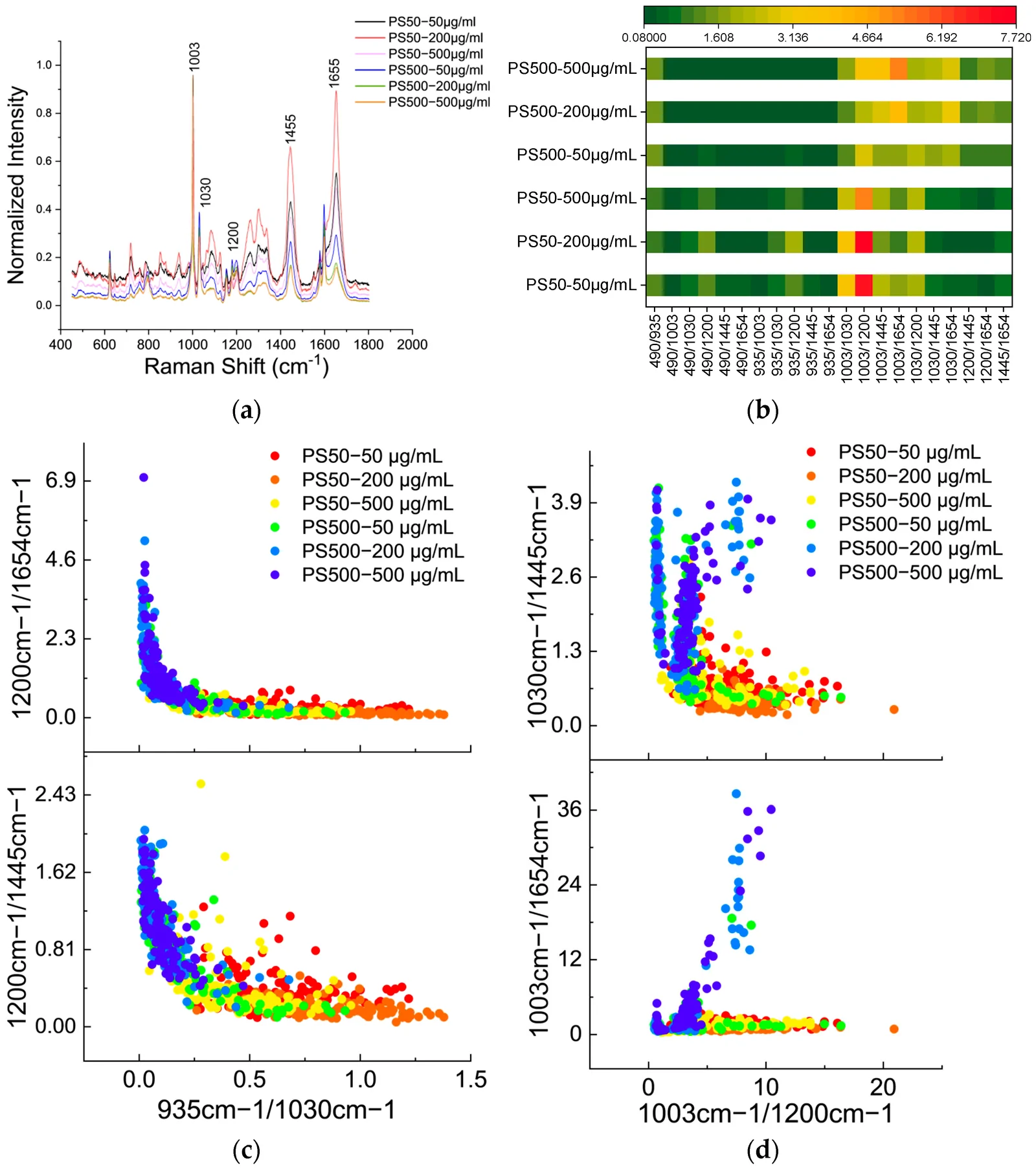
Averaged Raman spectra of Caco-2 cells treated with plain polystyrene nanoplastics (PS50 nm and PS500 nm) after 24 h treatment at different concentrations, 50, 200, and 500 µg/mL (**a**). Heat map of various peak ratios (**b**). Biaxial distribution of the Raman peak intensity ratios (**c**,**d**).

Under our current treatment conditions on polystyrene particles PS50, PS500 (Figure 1), and PSNH2 (Figure 3), Caco-2 cells suffer oxidative stress, but most of the cell viability is above 85%. Applying a non-destructive cell-based analytical instrument technique, such as Raman spectroscopy, to evaluate the biochemical changes (protein, carbohydrates, lipids, nucleic acids) of exposure cells provides additional information on cellular response. To detect the spectral markers of cells treated with nanoparticles, a comparison between the average of the normalized Raman spectra (in fingerprint range 450–1800 cm^−1^) of Caco-2 cells is presented in Figure 4a. The characteristic spectral peaks that appear in Figure 4a can be attributed to specific biomolecules, according to previously published studies [22,44,48]. The main spectral differences between different treatments are related to a large contribution of the amide III, CH_2_ twisting (l.), a combination of CH_3_ deformation and CH_2_ wagging (p.), CH_2_ scissoring, and amide I.

The major Raman peaks in all spectra are related to the protein vibrational modes, specifically amide I (at about 1655 cm^−1^), amide III (at about 1260–1300 cm^−1^), CH (at 1320 and 1340 cm^−1^) and CH_2_ (at 1445 cm^−1^) bending, the phenylalanine ring-breathing mode (at 1003 cm^−1^), and CC (at 1060, 1082, 1580–1600 cm^−1^) and CN (1060, 1082 and 1130 cm^−1^) stretching modes. The amide I band, arising primarily from C=O stretching of the peptide backbone, is sensitive to protein secondary structure—shifts or intensity changes in this band are indicative of protein unfolding or conformational changes, which are well-established consequences of oxidative modification of proteins by ROS. The amide III band, reflecting N-H bending and C-N stretching, similarly reports on changes in protein secondary structure and has been associated with altered α-helix to β-sheet ratios in oxidatively stressed cells. Changes in CH_2_ bending and scissoring modes (1445 cm^−1^) reflect alterations in lipid acyl chain structure and are particularly relevant in the context of lipid peroxidation, a hallmark of ROS-induced membrane damage in which unsaturated fatty acid chains undergo oxidative degradation, altering their vibrational signature. The phenylalanine ring-breathing mode at 1003 cm^−1^ serves as an internal reference for total cellular protein content, and relative changes in peak ratios involving this band across treatment conditions suggest concentration-dependent shifts in the overall protein-to-lipid balance of the cell, consistent with proteolytic activity and membrane disruption during early apoptosis. The CH stretching modes at 1320 and 1340 cm^−1^ reflect contributions from both proteins and nucleic acids, and their alteration under NP exposure may reflect DNA backbone reorganization or chromatin condensation, processes known to accompany mitochondria-dependent apoptotic pathways [49,50,51,52].

The heat map in Figure 4b presents the variations in Raman spectral peak ratios across different treatment conditions of Caco-2 cells exposed to microplastics. The *x*-axis represents the Raman peak ratio values at various Raman shifts, while each row represents a different treatment condition (e.g., different plastic concentrations), highlighting how the biochemical compositions of the exposure cells change due to microplastic exposure. The heterogeneity in peak intensities across different treatments suggests differential responses of Caco-2 cells to varying microplastic exposures.

The heat map shows distinct spectral differences between PS50 and PS500 microplastics. The cells treated with PS500 particles appear to induce more prominent peak ratio changes (see the intensity color gradient changes) with some regions changing from green to yellow and orange, suggesting a stronger biochemical response at specific Raman bands. The ratios that include the 1003 cm^−1^ peak (phenylalanine), show the most changes. Peak ratios with the most changes are used for biaxial ratio plots, as shown in Figure 4c,d. To reveal variances between cells under different treatments, the distribution of cells is visualized using a stacked scatter plot, where the *x*-axis represents one intensity ratio, while the *y*-axes display other Raman intensity ratios. Figure 4c,d illustrate that size-dependent clusters have formed, suggesting that nanoparticles induce cellular stress. However, the extent of this stress varies depending on particle size, which was not clearly observed using the conventional biology assay (Figure 1).

**Figure 5:**
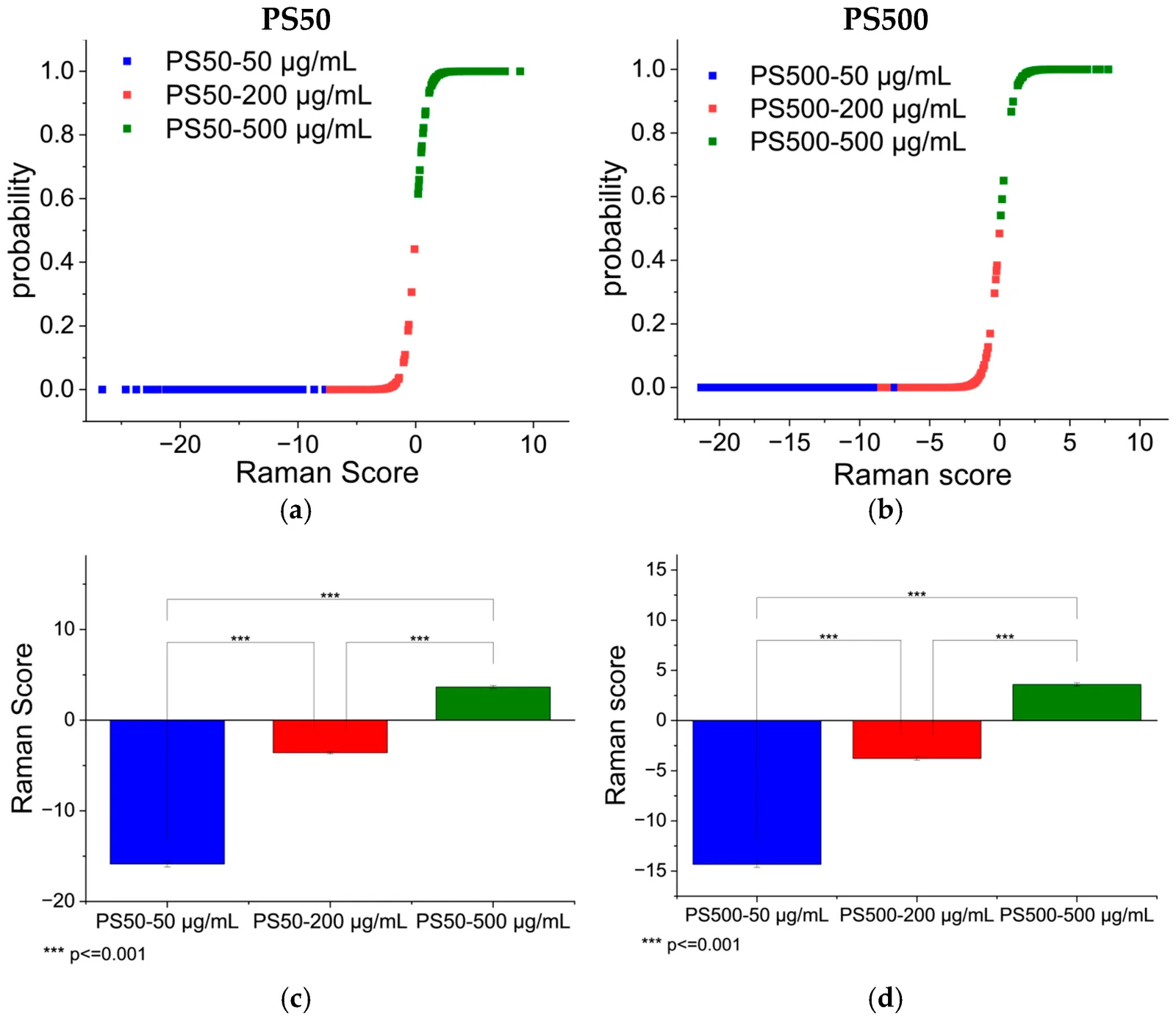

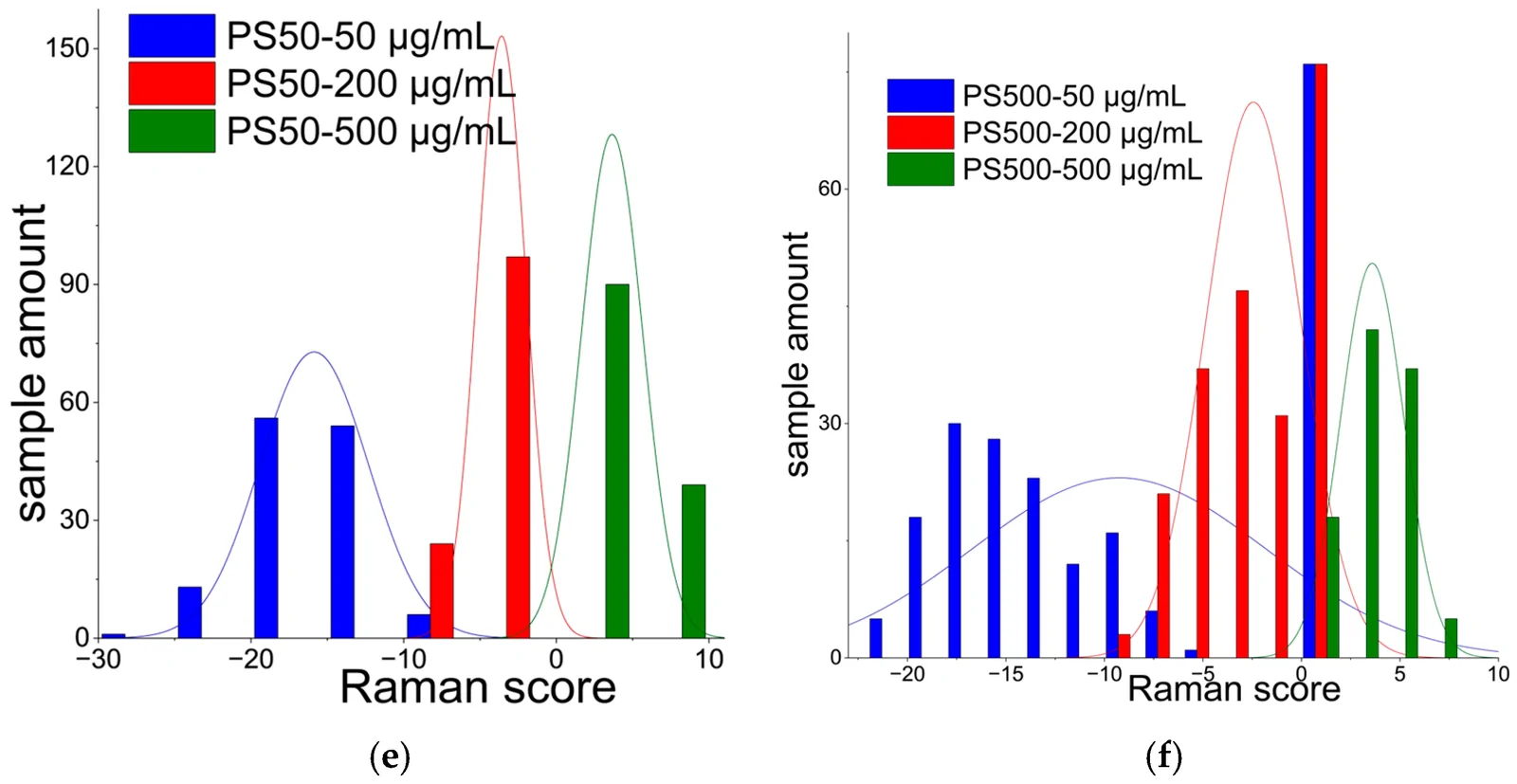
Raman score shifts for Caco-2 cells treated by PS50 (**a**–**c**) and PS500 (**d**–**f**) at 50, 200, and 500 µg/mL, respectively. The Raman score was first created by the generation of posterior probability from LDA (**a**,**b**) The posterior probability was then transformed to Raman score (**c**,**d**). Last, the histogram of Raman scores was created for the three concentration groups (**e**,**f**).

To assess a quantitative index for statistical analysis, posterior probability from LDA was used to characterize this process (each spectrum has one probability value). To better assess the cells’ Raman responses to nanoparticle treatment among the three concentrations (50 µg/mL, 200 µg/mL, 500 µg/mL), the probability values were linearized from PCA-LDA along a logistic function for scoring the degree of the individual cell response to PS50 treatment (Figure 5a). LDA classification under leave-one-out cross-validation yielded accuracies of 98.68%, 95.81%, and 88.33% for PS50, PS500, PS100NH2 conditions respectively, indicating that the model generalizes beyond the training data. The Raman score in cells treated with PS50 was significantly increased upon concentration, from −15.86 (50 µg/mL) to 3.64 (500 µg/mL) (Figure 5a–c). Similarly, 500 µg/mL treatment with PS500 particles remarkably increased the score from −14.3 (50 µg/mL) to 3.59 (500 µg/mL). (Figure 5d–f). The shift from negative to positive Raman score values with increasing concentration reflects a progressive biochemical transition from a control-like to an NP-treated-like spectral state at the single-cell level. The distributions of Raman scores assigned to individual cells under each treatment concentration, shown as histograms in Figure 5e,f, reveal the heterogeneity of the cellular responses within the population. This cell-level information is an important feature of the scoring approach, which uses statistical analysis of noninvasive Raman spectroscopy measurements to capture heterogeneity that traditional cell viability assays cannot detect. Given the magnitude of changes in Raman scores, it suggests that nanoparticles, regardless of size, significantly impact the molecular composition of Caco-2 cells. The Raman score result for PS100NH2 can be found in Figure S3. The Raman score was then used for a correlation plot between the biological assay and Raman results.

**Figure 6:**
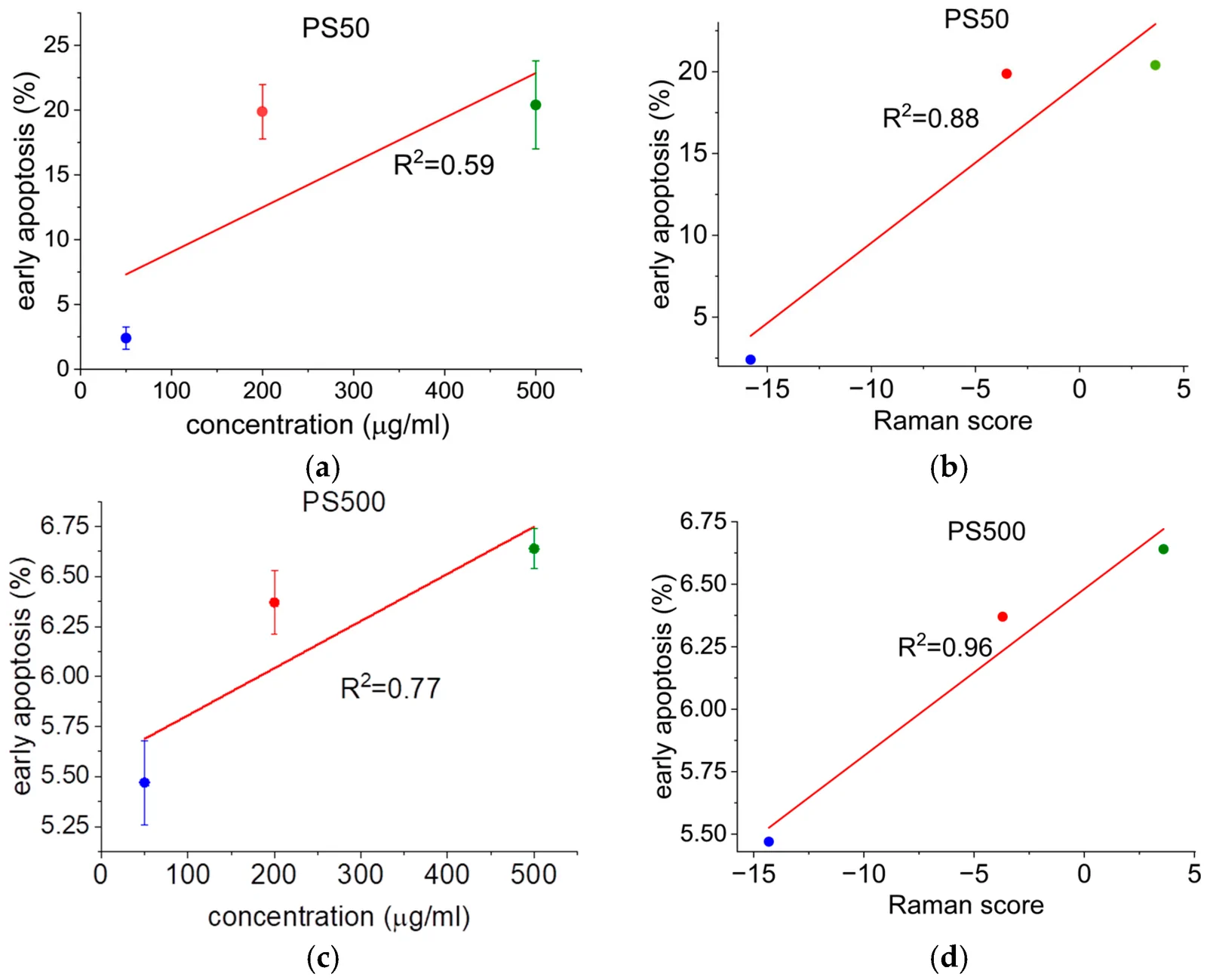

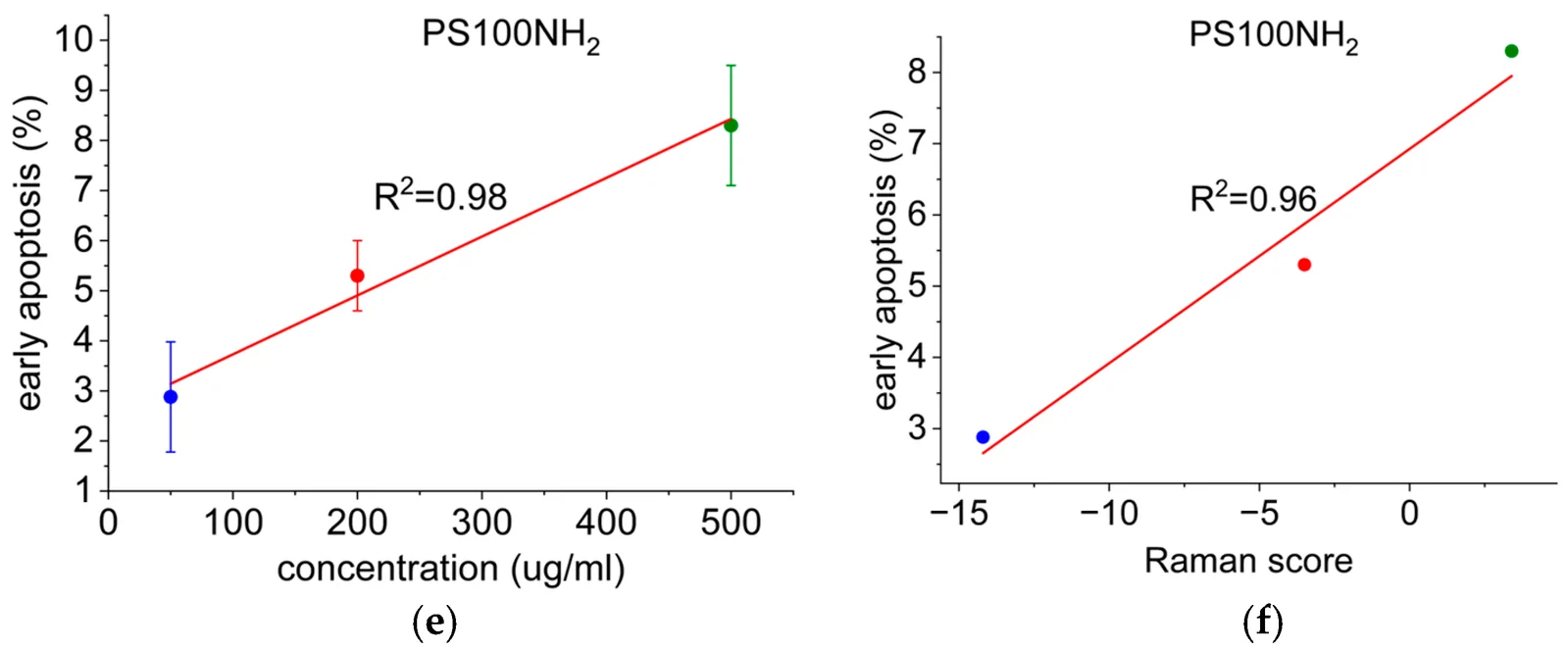
Correlation between concentration of nanoparticles and early apoptosis (**a**,**c**,**e**), and correlation between Raman score and early apoptosis (**b**,**d**,**f**). Each point represents the mean value of three independent biological replicates (*n* = 3).

First, the correlation between early apoptosis and concentration of nanoparticles was obtained for each particle size. Figure 6a,c,e illustrate the relationship between different concentrations of PS50, PS500, and PS100NH2 and the percentage of early apoptotic cells. A positive correlation (R^2^ = 0.59, 0.77, and 0.98, respectively) is observed, suggesting that higher concentrations of nanoparticles are associated with an increase in early apoptosis. Although the overall R^2^ value indicates moderate correlation strength, this trend suggests that even larger polystyrene microparticles, which do not significantly impact overall cell viability, may still induce early apoptotic changes at higher concentrations. Then the Raman score was correlated to the percentage of early apoptosis, with R^2^ = 0.88, 0.96, and 0.96 in response to PS50, PS500, and PS100NH2, respectively, as shown in Figure 6b,d,f.

### 3.3. Machine Learning Clustering

**Figure 7:**
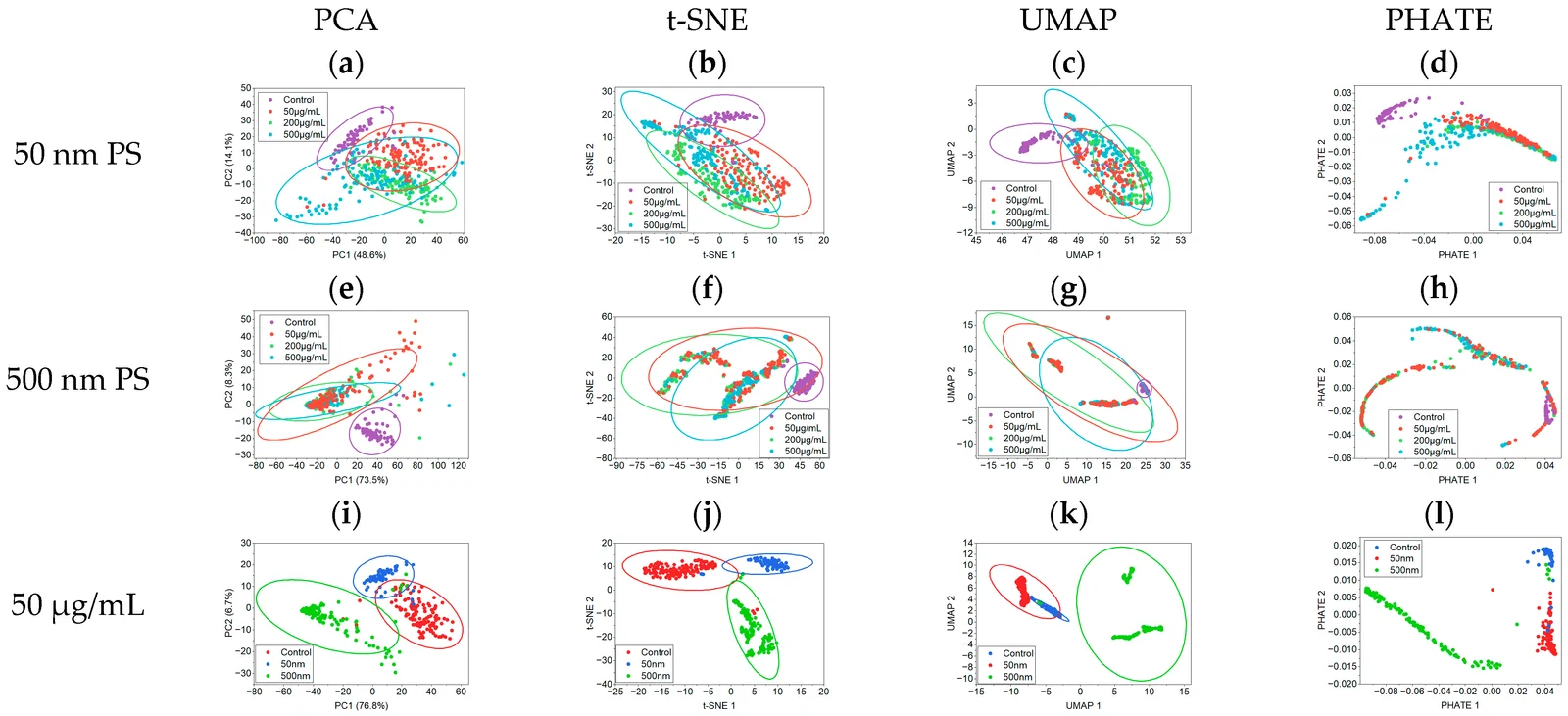

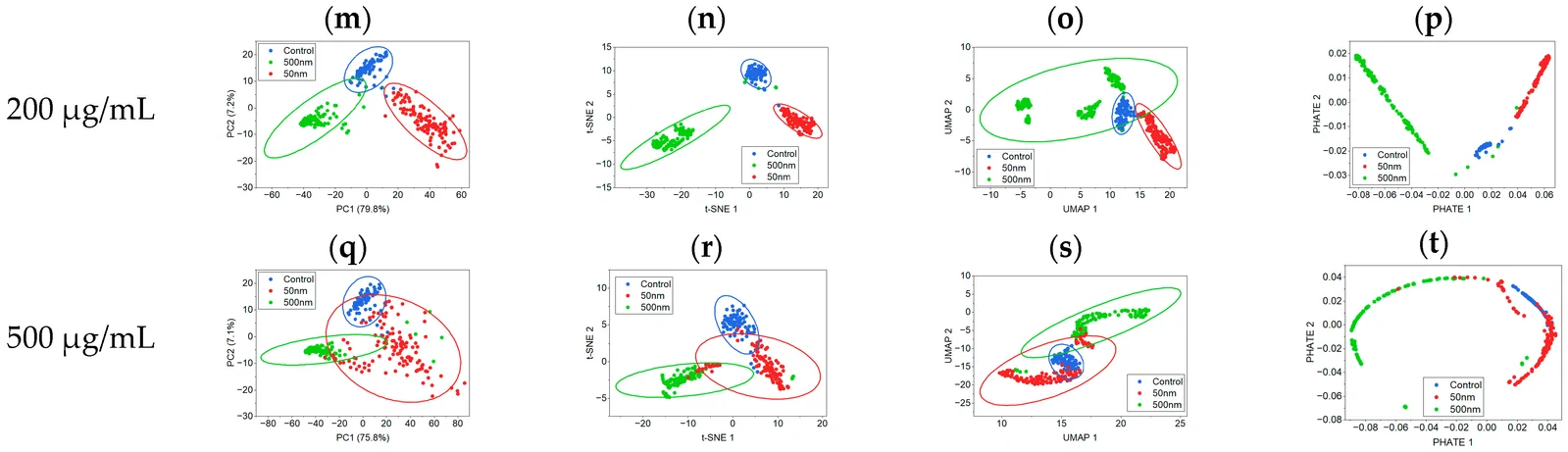
Data visualization of Raman spectra using PCA, t-SNE, UMAP, and PHATE. Raman spectra obtained from cells treated with the two sizes of polystyrene particles for 24 h at three concentrations. The results are displayed either by PS size (PS50 (**a**–**d**); 500 (**e**–**h**)) or by concentration (50 µg/mL (**i**–**l**); 200 µg/mL (**m**–**p**); 500 µg/mL (**q**–**t**)). Each dot represents one Raman spectrum from 600 to 1800 cm^−1^.

Raman spectrum data were processed via Python scripts which employ four algorithms that reduce and categorize the data to identify related components within a dataset. The preliminary Raman measurements of Caco-2 cells treated with various concentrations of nanoparticles (corresponding to the results shown in Figure 1) were performed. The Raman spectra data were visualized using PCA, t-SNE, PHATE, and UMAP, as shown in Figure 7.

The qualitative analysis of the nanoparticle treatment showed moderate correlation clustering for four groups across two sizes of PS NPs (Figure 7a–h). In Figure 7i–t, size-dependent clustering for each concentration of nanoparticles is shown. Clear differentiation demonstrates that each size of particles makes detectable biochemical changes. Quantitative analysis of these data would ordinarily be performed using mutual information estimation [53] to determine the Raman shift samples most associated with the treatment concentration. For this study, normalized mutual information was used to determine the hyperparameters used. Additionally, a silhouette score was calculated. Both values and the associated hyperparameters are in Table S2.

The highest NMI calculated was 0.929 and corresponds to the PHATE analysis on the different sizes of nanoparticles at 200 µg/mL (Figure 7p). The highest silhouette score calculated was 0.762 for the same data set but using t-SNE for the analysis (Figure 7n). There were consistently higher NMI and silhouette scores when comparing the different sizes at the same concentration than the same size at different concentrations.

### 3.4. Antioxidant Production

After toxicity conditions had been established, the use of antioxidants as protective agents was tested. The first step was to determine the optimal concentration of resveratrol (RES) to use as a protective agent against PS NPs.

**Figure 8:**
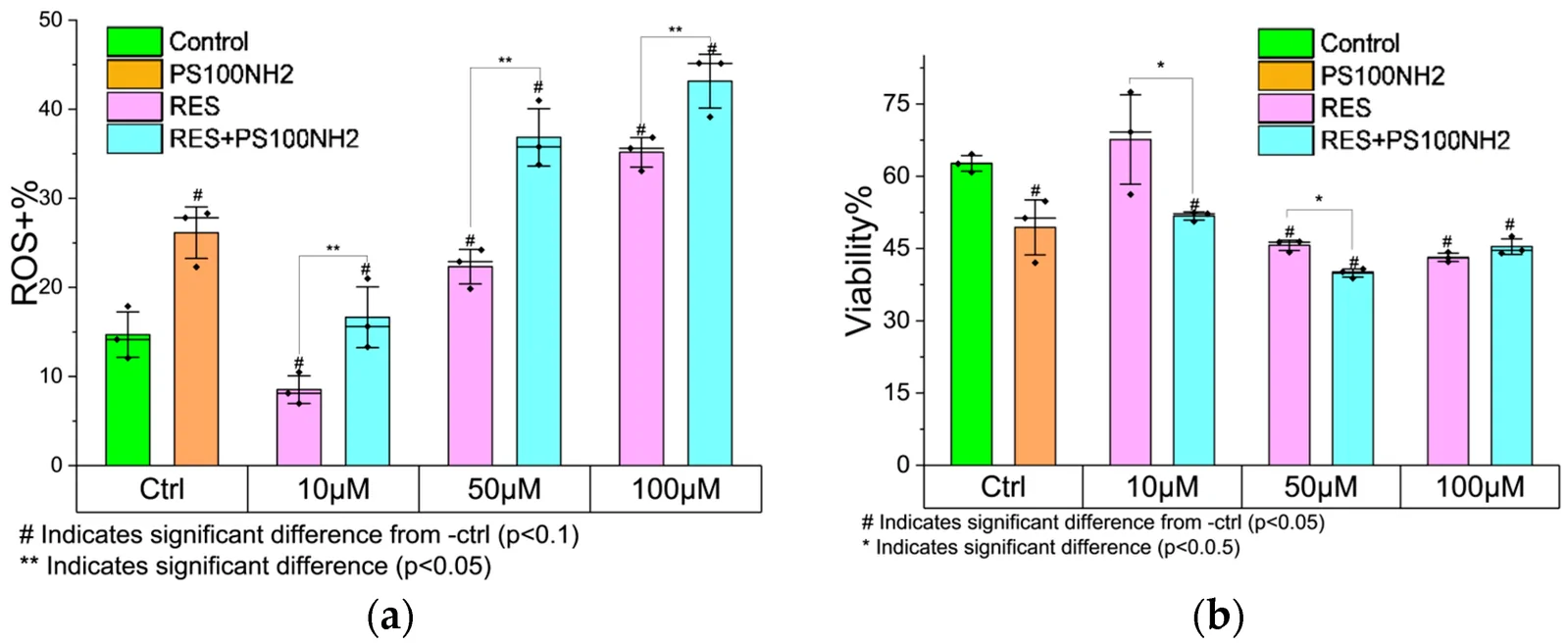
Effect of resveratrol concentration on ROS (**a**) and viability (**b**) of Caco-2 cells treated with 100 nm amine-modified polystyrene nanoparticles (PS100NH2) for 24 h after the pretreatment of different resveratrol concentrations for 24 h: 10 µM, 50 µM, 100 µM. Controls: negative control (Ctrl, the cells without any treatment), positive control (PS100NH2) only. PS100NH2 concentration is 200 µg/mL. ** indicates significant difference (*p* < 0.05). * indicates significant difference (*p* < 0.1). # indicates significant difference from Ctrl group (*p* < 0.1). For each treatment three independent biological replicates (*n* = 3) were measured.

The graphs in Figure 8a,b illustrate the interesting results of this experiment, which used a pretreatment of three concentrations of resveratrol (RES: 10 µM, 50 µM, 100 µM) for 24 h prior to a 24 h treatment of nanoparticles PS100NH2. These data suggest that the protective potential of resveratrol is highly dependent on RES concentration. The samples with only RES added show an increase in ROS response directly correlated to RES concentration, but only the 10 µM RES group shows a decrease in ROS relative to the control group. However, when resveratrol concentration is increased to 50 µM or 100 µM, the ROS produced by RES + PS100NH2 are higher than the group treated with just PS100NH2.

**Figure 9:**
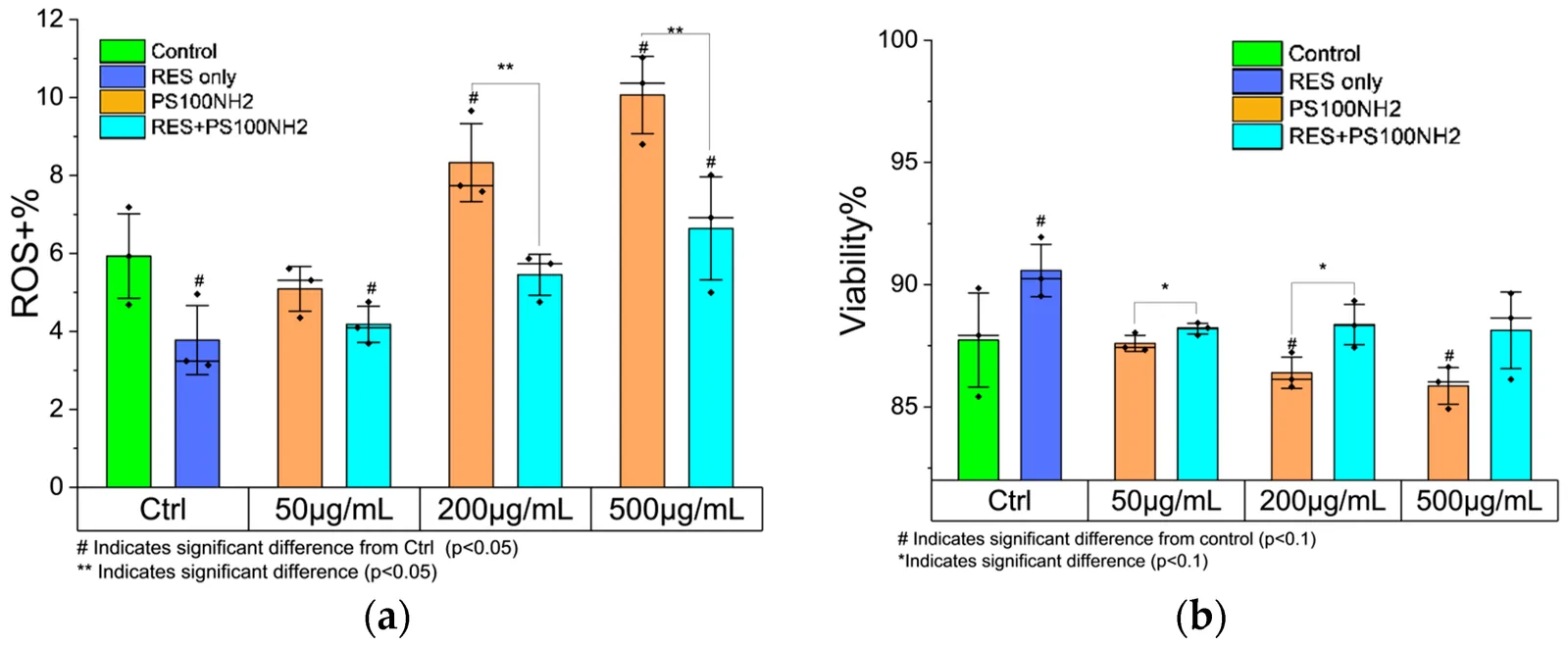
Effect of the concentration of amine-modified polystyrene nanoplastics (PS100NH2) on ROS (**a**) and viability (**b**) of Caco-2 cells. The cells pretreated with 10 µM resveratrol for 24 h, followed by 24 h treatment with different PS100NH2 concentrations: 50 µg/mL, 200 µg/mL, 500 µg/mL. Control: cells without any treatment, or cells treated with resveratrol (RES) only. ** indicates significant difference (*p* < 0.05). * indicates significant difference (*p* < 0.1). # indicates significant difference from Ctrl group (*p* < 0.1). For each treatment three independent biological replicates (*n* = 3) were measured.

**Figure 10:**
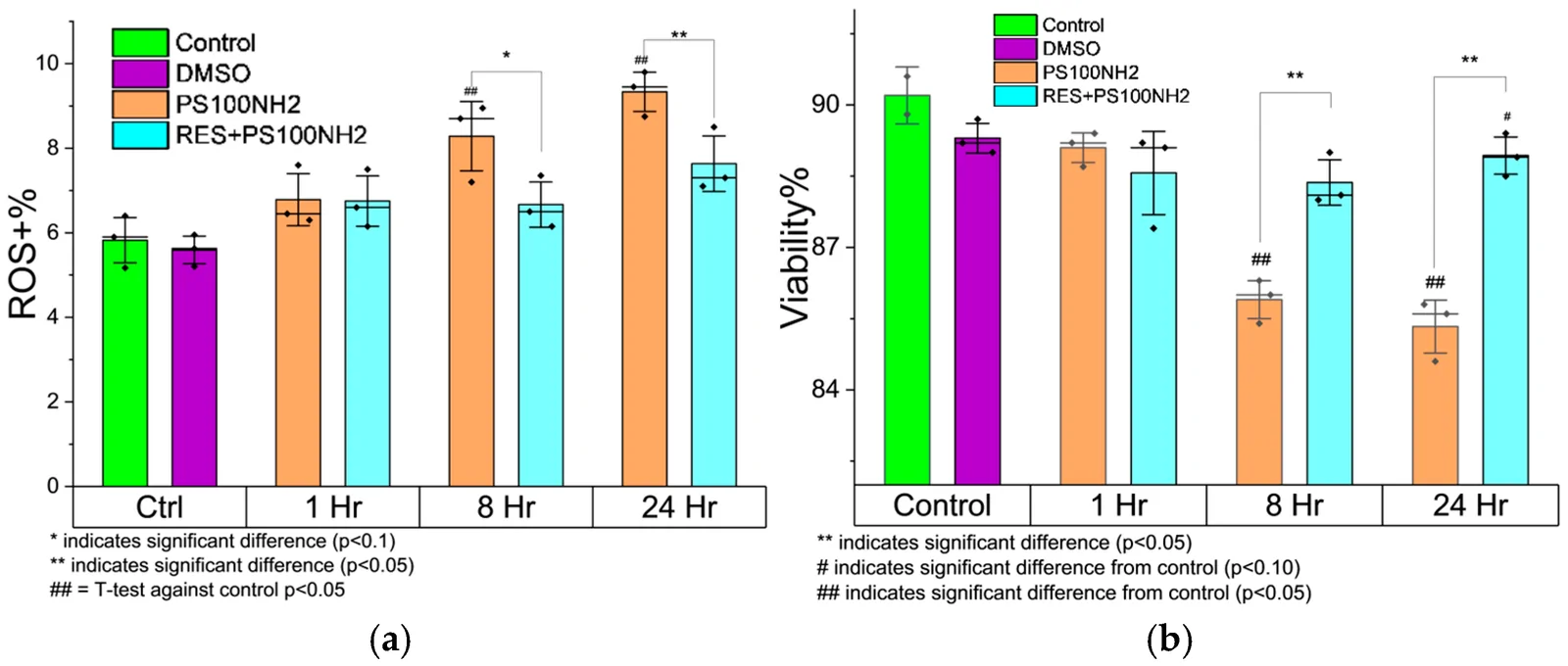
Time-dependence of amine-modified polystyrene nanoplastics (PS100NH2) treatment on ROS (**a**) and viability (**b**) of Caco-2 cells. The cells pretreated with 10 µM resveratrol for 24 h, followed by different PS100NH2 treatment time: 1 h, 8 h, 24 h. Co Control: cells without any treatment, or cells treated with DMSO only. ** indicates significant difference (*p* < 0.05). * indicates significant difference (*p* < 0.1). # indicates significant difference from Ctrl group (*p* < 0.1). ## indicates significant difference from Ctrl group (*p* < 0.05). For each treatment three independent biological replicates (*n* = 3) were measured.

After the optimal resveratrol treatment concentration (10 µM) was determined, protective pretreatments with resveratrol were applied to cells followed by amine-modified PS100NH2 at various concentrations and treatment times. Figure 9 shows the protective effect of resveratrol at different concentrations of NPs. At a particular concentration of PS100NH2 (e.g., 50 µg/mL), ROS of cells treated with RES + PS100NH2 are decreased, in comparison to PS100NH2 only. This trend in ROS change is consistent across three PS100NH2 concentrations (50 µg/mL, 200 µg/mL, 500 µg/mL), similar to the trend in viability. As for the time-dependence of the PS100NH2 treatment, Figure 10 shows that the longer treatment time, the higher ROS or lower viability. However, most RES-involved groups show the protective effect by reducing ROS (increasing viability), which most distinctly occurred at 24 h.

## 4. Discussion

While some nanoparticles appear to have minimal impact on overall cell viability, a more detailed analysis of apoptosis profiles reveals potential early apoptotic changes. This suggests that certain nanoparticles may induce cellular stress responses that do not immediately result in cell death but may still disrupt cellular homeostasis [47,54]. A key novelty of this work lies in applying the Raman score, a continuous logistic index derived from PCA-LDA posterior probabilities to nanoplastics toxicology for the first time. The magnitude of the score reflects the degree of that shift, making it a graded rather than binary measure of cellular perturbation. The progressive shift from strong negative scores at 50 µg/mL to near-zero or positive scores at 500 µg/mL is therefore interpretable as a genuine, concentration-dependent biochemical transition. Unlike discrete apoptosis categories from flow cytometry, this score captures graded biochemical change from population cells and correlated with early apoptosis across all three particle types (R^2^ = 0.88, 0.96, and 0.96 for PS50, PS500, and PS100NH_2_, respectively), demonstrating its potential as a predictive marker for apoptotic progression. The correlations presented in Figure 6 are based on three concentration points per particle type, which reflect the experimental design rather than a statistical limitation per se. Each point represents the mean value of three independent biological replicates (*n* = 3). The correlations observed (R^2^ = 0.88–0.98) are supported by the consistency of the trend across all three particle types and by the large number of individual Raman spectra contributing to each Raman score data point (~130 spectra from 11–14 cells per condition), which provides substantial spectral statistical power even when the number of concentration points is limited (e.g., three concentrations used here). Expanding the number of tested concentrations in future work would allow for more robust regression analysis and stronger confidence in the predictive relationship between Raman score and apoptosis.

The systematic comparison of PCA, t-SNE, UMAP, and PHATE on the same dataset further distinguishes this work from prior Raman-based oxidative stress studies, as it reveals size-dependent biochemical clustering that was not detectable by conventional viability assays. It should be acknowledged that the concentrations used in this study are higher than current environmental exposure estimates; however, they fall within the range commonly used in most in vitro nanotoxicology studies and were selected to establish clear dose-response relationships. Whether similar biochemical signatures emerge at environmentally relevant concentrations remains an important question for future work. This study was also intentionally limited to polystyrene particles, which served as a well-controlled model system for developing and validating the Raman-based analytical framework. Environmentally relevant polymers such as PE, PP, PET, PVC, and nylon differ in surface chemistry, density, and degradation behavior, and may induce distinct biochemical responses in intestinal epithelial cells. Extending this Raman spectroscopy and machine learning approach to these polymer types is an important next step, and the methodology established here provides a direct template for doing so. Given that Raman spectroscopy is a tool used to obtain a chemical signature of a sample, further Raman analysis should be performed to deepen understanding of the biochemical signature of a cell and how that signature is impacted by overall cell health following chronic exposure to nanoplastics.

We can interpret the Raman spectral changes across treatment conditions as NP-induced oxidative stress and apoptosis. The changes in amide I and amide III bands, which are induced by different concentrations, are consistent with progressive oxidative modification of cellular proteins. This is particularly significant because protein oxidation is not only a downstream marker of ROS damage but an active cause of mitochondrial dysfunction, a process that leads the cell toward apoptosis. The changes in CH_2_ bending modes reflect lipid peroxidation of membrane phospholipids, which damages membrane integrity, increases permeability, and facilitates the release of cytochrome c from the mitochondrial inner membrane. This process is a key trigger of caspase-3 activation and the intrinsic apoptotic cascade [55,56]. There is a size-dependent difference in these spectral signatures, which suggests that bigger particles might produce a stronger lipid peroxidation response, possibly because of a larger surface area to interact with the plasma membrane. On the other hand, smaller PS50 particles appear to exert greater effects on overall cell viability at higher concentrations through a distinct mechanism. We want to be clear that without targeted molecular assays, this mechanistic interpretation of the spectral data is necessarily inferential. However, the Raman score is correlated with early apoptosis, which is in line with the literature on NP-induced ROS-mediated cell death. Future work combining Raman spectroscopy with targeted biochemical assays such as lipid peroxidation assays (MDA/4-HNE), protein carbonyl detection, and caspase-3 activity measurements would allow direct validation of this spectral–mechanistic linkage.

The resveratrol data indicate that its protective potential is highly concentration dependent. At 10 µM, resveratrol reduced ROS in cells treated with PS100NH2, whereas higher concentrations (50 µM and 100 µM) appeared to exacerbate ROS rather than mitigate it. This is consistent with findings from other studies showing that higher concentrations of resveratrol can trigger apoptosis and senescence pathways in cancer cells [35], which complicates its concentration-dependent protective role. At the optimal 10 µM concentration, resveratrol consistently reduced ROS and improved viability across all tested PS100NH2 concentrations, with the most pronounced protective effect occurring at 24 h of treatment. It should be noted, however, that these findings are based on an in vitro model, and the results may not necessarily translate to in vivo conditions. Also, we will further evaluate this Raman–machine learning approach in assessment of cytotoxicity and apoptosis in other cell lines (e.g., human alveolar basal epithelial cells A549) or other antioxidants (e.g., Curcumin, Vitamin C).

Taken together, these findings demonstrate the utility of combining Raman spectroscopy and machine learning to characterize the cytotoxic effects of nanoplastics at a molecular level. Machine learning algorithms, such as PCA, t-SNE, UMAP, and PHATE, provided valuable insights into data visualization and facilitated interpretation of complex spectral datasets. The correlation between Raman score and early apoptosis, along with the size-dependent clustering patterns, supports the use of Raman spectroscopy as a sensitive, noninvasive tool for monitoring nanoparticle-induced cellular damage. Future work incorporating additional machine learning algorithms could further identify the Raman shifts most associated with specific treatment conditions, strengthening the quantitative power of this approach.

## 5. Conclusions

In summary, this research delves into the intricate relationship between nanoplastics (NPs) and microplastics (MPs) and their effects on mammalian intestinal cells, shedding light on a pressing ecological and health concern. Through the experiments described, the distinct impacts of NPs and MPs on cellular reactive oxygen species (ROS) response and apoptosis were found, underscoring the necessity for a nuanced understanding of nanoplastic pollutants. Leveraging resveratrol as a protective agent, the study unveiled the complex interplay between antioxidants and nanoplastic-induced cytotoxicity. Other studies have shown that higher concentrations of resveratrol trigger apoptosis and senescence pathways in cancer cells, which complicates the concentration-dependent protective effect of resveratrol [35].

The incorporation of Raman spectroscopy data offers further avenues for correlating biological responses with molecular changes induced by nanoplastics exposure. Machine learning algorithms, such as PCA, t-SNE, UMAP, and PHATE, offer valuable insights into data visualization, facilitating the interpretation of complex datasets and correlations among treatment conditions. Raman spectroscopy, coupled with machine learning analysis, provides additional layers of understanding, along with correlation clustering that suggest avenues for future exploration.

Overall, this research contributes to our methodologies for testing toxic and protective agents in vulnerable human cells as well as our understanding of the biological impacts of nanoplastic pollution. For example, studying the potential correlation between molecular changes measured by Raman spectroscopy and apoptosis signaling pathways (NF-kB, MAPK, Nrf2) would provide additional understanding of the mechanisms of genotype responses from nano- and microplastic-exposed cells.

Further investigations into nanoplastic-induced cytotoxicity and the efficacy of protective agents are imperative for devising effective mitigation strategies and safeguarding environmental and human health in the face of escalating human-caused pollution.

## Data Availability

Data will be made available on request.

## Supplementary Materials

The following supporting information can be downloaded at: https://www.mdpi.com/article/10.3390/s26175375/s1, Figure S1: Results of dynamic light scattering. Each type of particle was incubated in completed media at concentration of 200 µg/mL for 24 h. Measurements were taken immediately upon preparation (0 h) and after 24 h of incubation. PS50 after 0 h (a), PS50 after 24 h (b), PS100NH2 after 0 h (c), PS100NH2 after 24 h (d), PS500 after 0 h (e), PS500 after 24 h (f); Table S1: Comparison of zeta potential before and after incubation. Each type of particle was incubated in completed media at a concentration of 200 µg/mL for 24 h. Measurements were taken immediately upon preparation (0 h) and after 24 h of incubation.; Figure S2: Apoptosis profile of Caco-2 cells treated with PS100NH2 after 24 h treatment at different concentrations: 50 µg/mL (a), 200 µg/mL (b), 500 µg/mL (c). These flow cytometry results are extended from Figure 3b; Figure S3: Raman score shifts of Caco-2 cells treated by PS100NH2 at 50, 200, and 500 µg/mL. The Raman score was first created by the generation of posterior probability from LDA (a). * *p* < 0.05. The posterior probability was then transformed to Raman score (b). Last, the histogram of Raman score was created across three concentration groups (c); Table S2: Hyperparameters used for Figure 7 and the calculated silhouette score and normalized mutual information (NMI) for each analysis; Figure S4: ROS and viability of Caco-2 cells treated with Resveratrol (RES) in different solvents and H_2_O_2_. Non-normalized ROS and Viability values obtained after 24-h treatment with RES, followed by 8-h treatment with 500 µM H_2_O_2_. Cells are either untreated (Ctrl, 1st green column) or treated with H_2_O_2_ only (red column) or pretreated with different concentrations of RES dissolved in solvents DMSO or NADES: 10 µM RES (orange and yellow columns 3 and 4), 50 µM RES (orange and yellow columns 5 and 6), 100 µM RES (orange and yellow columns 7 and 8). Columns are numbered from left to right. *t*-test performed between each solvent used for each RES concentration, and against negative control. ** indicates significant difference (*p* < 0.05). # indicates significant difference from Ctrl group (*p* < 0.1). ## indicates significant difference from Ctrl group (*p* < 0.05) (*n* = 3).
