# Supplementary material for: Probe Cytotoxic and Oxidative Stress Effects of Nanoplastics on Caco-2 Cells: Insights from Raman Spectroscopy and Machine Learning
Source: Sensors (Basel). 2026 Aug 25;26(17):5375. doi: 10.3390/s26175375 (PMC13568252; doi:10.3390/s26175375)
Supplement: Supplementary file 1 [file sensors-26-05375-s001.zip › sensors-4451107-supplementary.pdf]

Supplementary Materials

Probe Cytotoxic and Oxidative Stress Effects of Nanoplastics on Caco-2 Cells: Insights from Raman Spectroscopy and Machine Learning

Bryan Gustafson [†], Negar Kosari [†], Emily Brothersen, Morgan Mosher and Anhong Zhou *

Department of Biological and Chemical Engineering, Utah State University, Logan, UT 84322-4105, USA

[†]These authors contributed equally to this work.

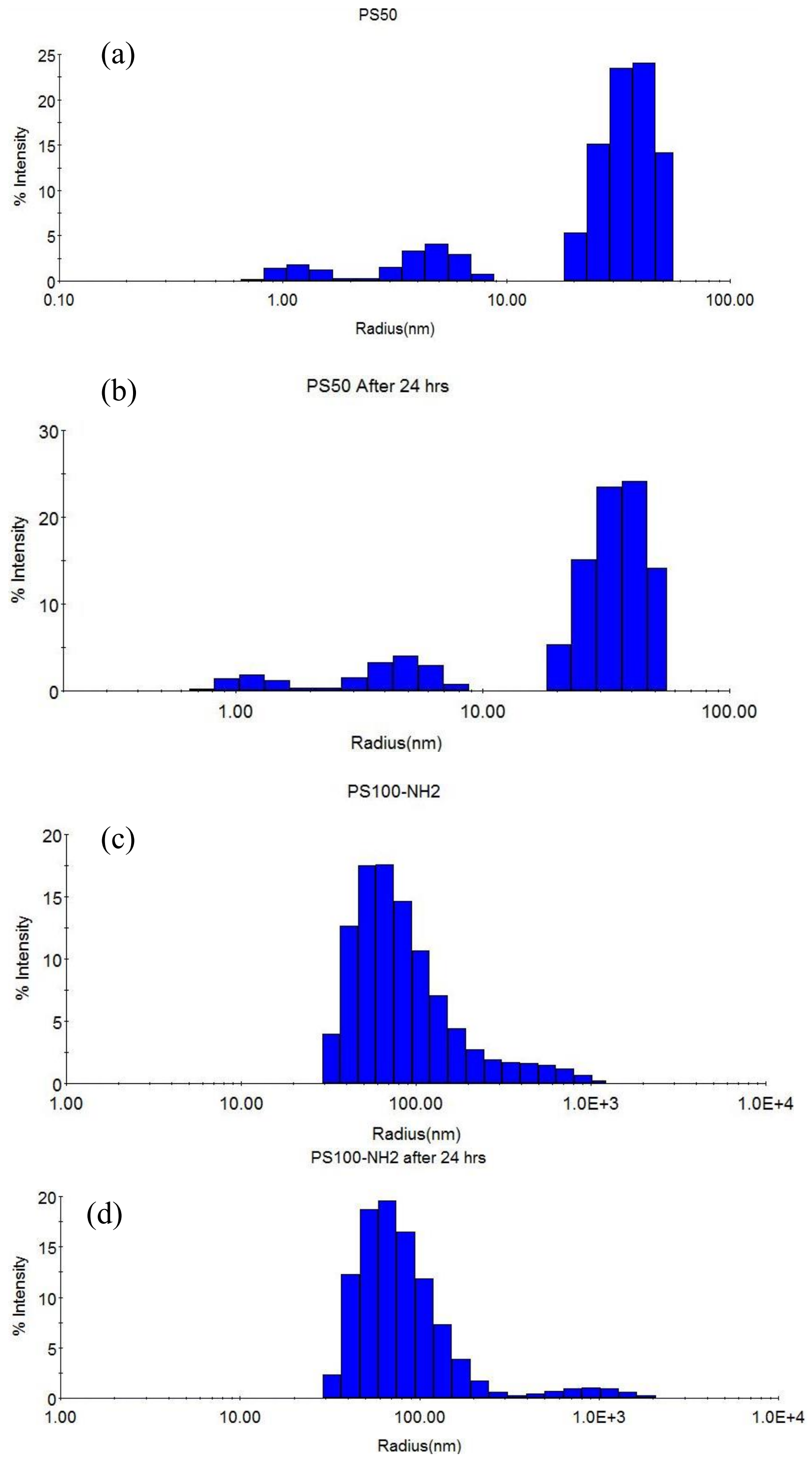

PS50
(a)
% Intensity
0
5
10
15
20
25
0.10
1.00
10.00
100.00
Radius(nm)
(b)
PS50 After 24 hrs
% Intensity
0
10
20
30
1.00
10.00
100.00
Radius(nm)
PS100-NH2
(c)
% Intensity
0
5
10
15
20
1.00
10.00
100.00
1.0E+3
1.0E+4
Radius(nm)
PS100-NH2 after 24 hrs
(d)
% Intensity
0
5
10
15
20
1.00
10.00
100.00
1.0E+3
1.0E+4
Radius(nm)

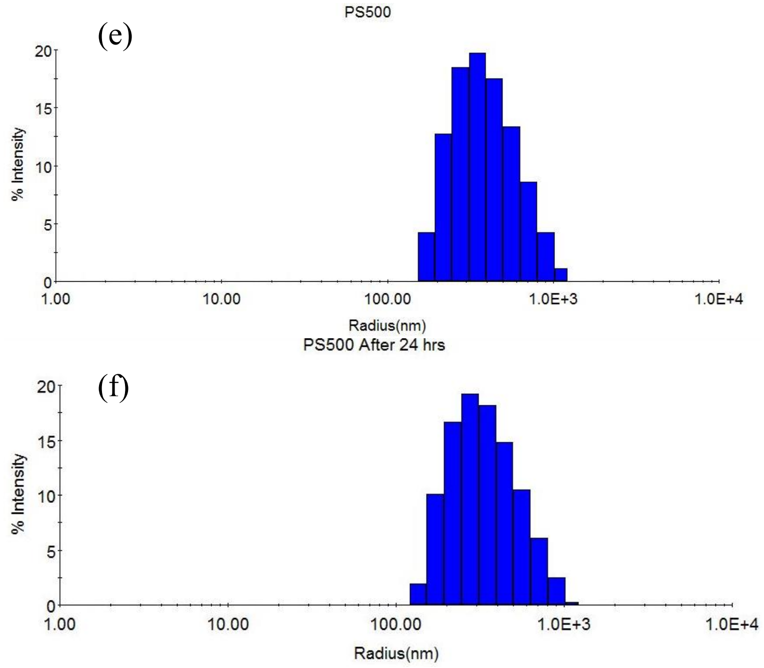

Figure S1. Results of dynamic light scattering. Each type of particle was incubated in completed media at concentration of 200 μg/ml for 24 hours. Measurements were taken immediately upon preparation (0 hours) and after 24 hours of incubation. PS50 after 0 hours (a), PS50 after 24 hours (b), PS100NH2 after 0 hours (c), PS100NH2 after 24 hours (d), PS500 after 0 hours (e), PS500 after 24 hours (f)

Table S1. Comparison of zeta potential before and after incubation. Each type of particle was incubated in completed media at a concentration of 200 μg/ml for 24 hours. Measurements were taken immediately upon preparation (0 hours) and after 24 hours of incubation.

Samples	Zeta potential at t=0	Zeta potential at t=24 hours
PS50	-48.35	-39.17
PS100NH2	13.71	21.69
PS500	49.24	58.82

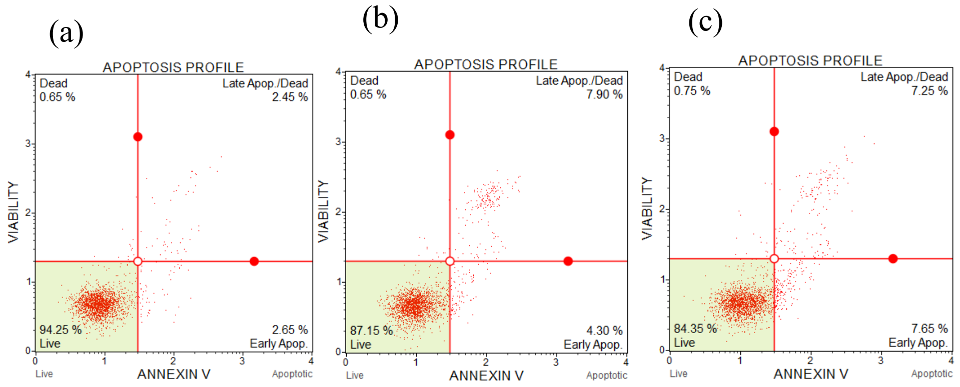

Figure S2. Apoptosis profile of Caco-2 cells treated with PS100NH2 after 24 hours treatment at different concentrations: 50 µg/mL (a), 200 µg/mL (b), 500 µg/mL (c). These flow cytometry results are extended from Fig. 3 (b).

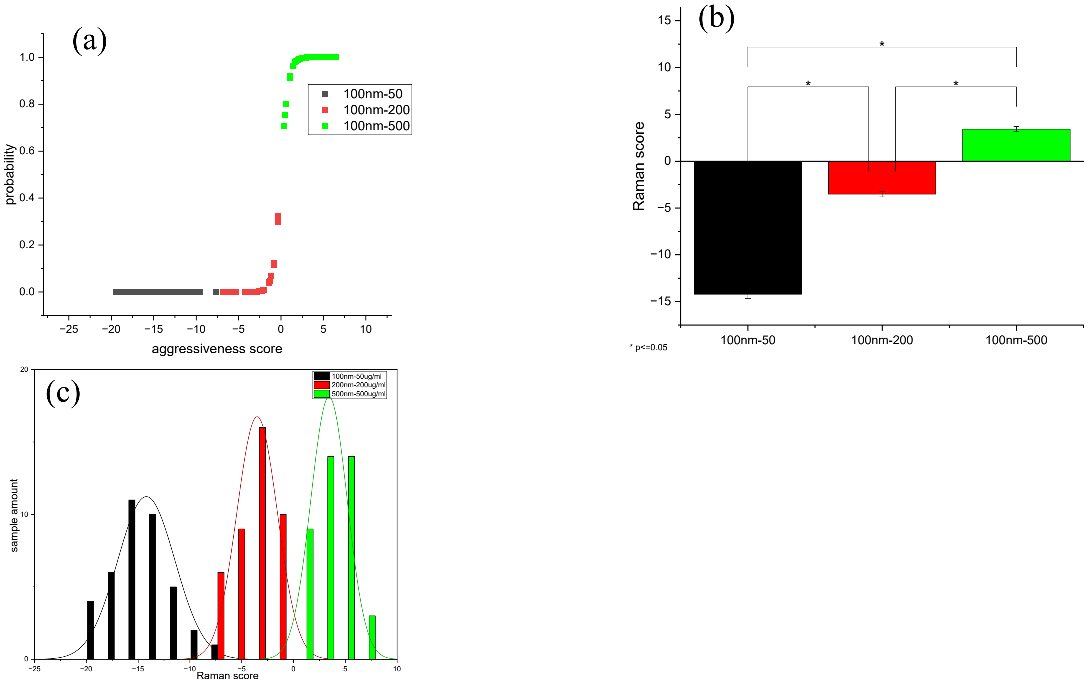

Figure S3. Raman score shifts of Caco-2 cells treated by PS100NH2 at 50, 200, and 500 µg/ml. The Raman score was first created by the generation of posterior probability from LDA (a). *$p < 0.05$. The posterior probability was then transformed to Raman score (b). Last, the histogram of Raman score was created across three concentration groups (c).

Table S2. Hyperparameters used for Figure 7 and the calculated silhouette score and normalized mutual information (NMI) for each analysis.

	**PCA**	**t-SNE**	**UMAP**	**PHATE**
Grid search parameters	n_components: 2	Perplexity: 10, 30, 50	Neighbors: 10, 15, 30<br>Min_dist: 0.1, 0.5	knn: 5, 15, 30
**50 nm PS**	Silhouette: 0.119<br>NMI: 0.229	Perplexity: 30<br>Silhouette: 0.096<br>NMI: 0.303	Neighbors: 15<br>Min_dist: 0.1<br>Silhouette: 0.056<br>NMI: 0.309	knn: 15<br>Silhouette: 0.041<br>NMI: 0.301
**500 nm PS**	Silhouette: 0.035<br>NMI: 0.344	Perplexity: 10<br>Silhouette: 0.116<br>NMI: 0.390	Neighbors: 10<br>Min_dist: 0.1<br>Silhouette: 0.073<br>NMI: 0.290	knn: 5<br>Silhouette: 0.046<br>NMI: 0.289
**50 μg/mL**	Silhouette: 0.503<br>NMI: 0.589	Perplexity: 30<br>Silhouette: 0.638<br>NMI: 0.854	Neighbors: 10<br>Min_dist: 0.1<br>Silhouette: 0.538<br>NMI: 0.858	knn: 30<br>Silhouette: 0.604<br>NMI: 0.714
**200 μg/mL**	Silhouette: 0.682<br>NMI: 0.845	Perplexity: 50<br>Silhouette: 0.762<br>NMI: 0.921	Neighbors: 10<br>Min_dist: 0.5<br>Silhouette: 0.337<br>NMI: 0.686	knn: 15<br>Silhouette: 0.691<br>NMI: 0.929
**500 μg/mL**	Silhouette: 0.439<br>NMI: 0.615	Perplexity: 50<br>Silhouette: 0.474<br>NMI: 0.655	Neighbors: 10<br>Min_dist: 0.5<br>Silhouette: 0.347<br>NMI: 0.596	knn: 5<br>Silhouette: 0.338<br>NMI: 0.505

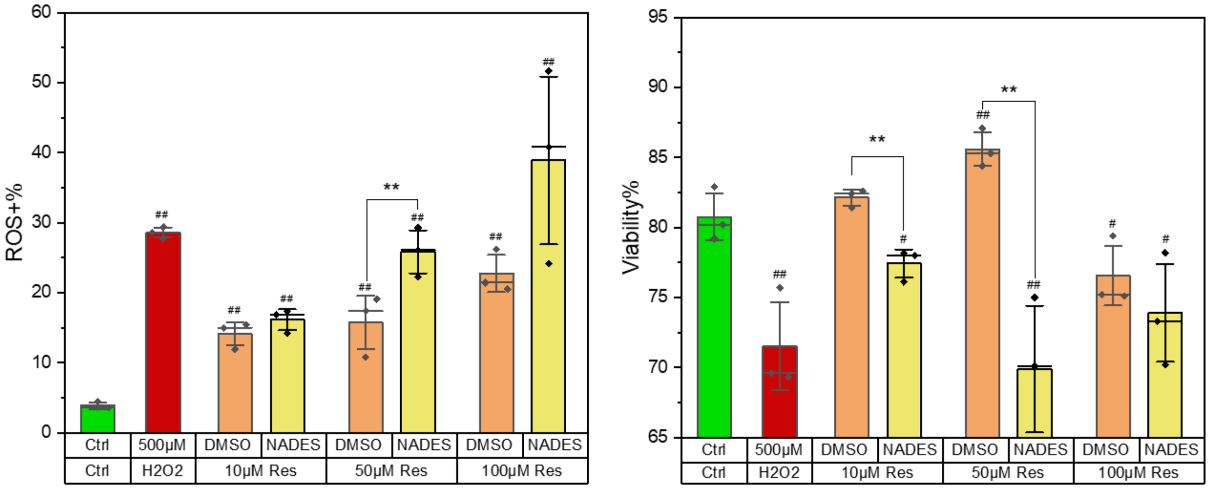

Figure S4. ROS and viability of Caco-2 cells treated with Resveratrol (RES) in different solvents and H_2O_2. Non-normalized ROS and Viability values obtained after 24-hour treatment with RES, followed by 8-hour treatment with 500 μM H_2O_2. Cells are either untreated (Ctrl, 1st green column) or treated with H_2O_2 only (red column) or pretreated with different concentrations of RES dissolved in solvents DMSO or NADES: 10 μM RES (orange and yellow columns 3 and 4), 50 μM RES (orange and yellow columns 5 and 6), 100 μM RES (orange and yellow columns 7 and 8). Columns are numbered from left to right. t-test performed between each solvent used for each RES concentration, and against negative control. ** indicates significant difference ($p<0.05$). # indicates significant difference from Ctrl group ($p<0.1$). ## indicates significant difference from Ctrl group ($p<0.05$) (n=3).
